# The Role of Selected lncRNAs in Lipid Metabolism and Cardiovascular Disease Risk

**DOI:** 10.3390/ijms25179244

**Published:** 2024-08-26

**Authors:** Anna Gluba-Sagr, Beata Franczyk, Aleksandra Rysz-Górzyńska, Robert Olszewski, Jacek Rysz

**Affiliations:** 1 Department of Nephrology, Hypertension and Family Medicine, Medical University of Lodz, 90-549 Lodz, Poland; beata.franczyk-skora@umed.lodz.pl (B.F.); jacek.rysz@umed.lodz.pl (J.R.); 2 Department of Ophthalmology and Visual Rehabilitation, Medical University of Lodz, 90-549 Lodz, Poland; mrs-89@o2.pl; 3 Department of Gerontology, Public Health and Didactics, National Institute of Geriatrics, Rheumatology and Rehabilitation in Warsaw, 02-637 Warsaw, Poland; robert.olszewski@me.com

**Keywords:** long noncoding RNA, lipid disorders, atherosclerosis

## Abstract

Lipid disorders increase the risk for the development of cardiometabolic disorders, including type 2 diabetes, atherosclerosis, and cardiovascular disease. Lipids levels, apart from diet, smoking, obesity, alcohol consumption, and lack of exercise, are also influenced by genetic factors. Recent studies suggested the role of long noncoding RNAs (lncRNAs) in the regulation of lipid formation and metabolism. Despite their lack of protein-coding capacity, lncRNAs are crucial regulators of various physiological and pathological processes since they affect the transcription and epigenetic chromatin remodelling. LncRNAs act as molecular signal, scaffold, decoy, enhancer, and guide molecules. This review summarises available data concerning the impact of lncRNAs on lipid levels and metabolism, as well as impact on cardiovascular disease risk. This relationship is significant because altered lipid metabolism is a well-known risk factor for cardiovascular diseases, and lncRNAs may play a crucial regulatory role. Understanding these mechanisms could pave the way for new therapeutic strategies to mitigate cardiovascular disease risk through targeted modulation of lncRNAs. The identification of dysregulated lncRNAs may pose promising candidates for therapeutic interventions, since strategies enabling the restoration of their levels could offer an effective means to impede disease progression without disrupting normal biological functions. LncRNAs may also serve as valuable biomarker candidates for various pathological states, including cardiovascular disease. However, still much remains unknown about the functions of most lncRNAs, thus extensive studies are necessary elucidate their roles in physiology, development, and disease.

## 1. Introduction

Cardiovascular disease (CVD) is one of the most common causes of mortality and morbidity globally [1]. According to estimations of the World Health Organisation (WHO), CVD is associated with 17.9 million deaths each year [2]. About 33% of these deaths are reported in people under the age of 70 years. Increased risk of CVD is associated with physical inactivity, unhealthy diet, and tobacco and alcohol use, leading to obesity and raised blood pressure, blood lipids, and glucose levels [2]. Moreover, the presence of some genetic alterations could affect the risk of CVD. Lipid metabolism disorders boost the risk of cardiometabolic disorders, such as type 2 diabetes and atherosclerosis, and therefore, the treatment of lipid disorders is the most frequent therapeutic strategy to decrease the incidence of CVD [1].

Various genetic mechanisms are involved in the regulation of lipid formation and metabolism and the modulation of cardiovascular risk. Recently, the role of noncoding RNA (ncRNA) in disease development has been suggested [3]. Noncoding RNAs can be divided into various categories, including microRNA (miRNA), long noncoding RNA (lncRNA), and circular RNA (circRNA) [4]. Numerous studies have suggested the involvement of miRNA in the development of atherosclerosis via endothelial dysfunction, lipid accumulation, inflammation, vascular angiogenesis, thrombosis, and calcification [5]. Their actions can be modified by long noncoding RNA that can either directly bind or interact with mRNA, which results in translational suppression or mRNA decay [6,7,8]. Moreover, lncRNAs can control the abundance of miRNAs, acting as molecular sponges [9]. Apart from miRNA, lncRNAs also bind with proteins, leading to the modification of their activity, stability, localisation, or sequestering [10]. Various studies focused on the identification of noncoding RNA molecules, which acting predominantly at a post-transcriptional level regulate the expression of genes involved in lipid metabolism and CVD [1,11]. The roles of miRNAs in the regulation of lipid metabolism and CVD have been well studied and widely described; however, there is only preliminary evidence concerning the contribution of lncRNAs to lipid homeostasis. Therefore, this review aimed to summarise the data concerning the impact of selected lncRNAs on the regulation of lipid metabolism, with a special focus on high-density lipoprotein (HDL).

## 2. Long Noncoding RNAs 

Only about 2% of the RNA generated in the process of the transcription of the mammalian genome encodes proteins [12,13]. Nowadays, we are gaining more and more information on the biological functions of lncRNAs. LncRNAs are a large class of alternatively spliced and polyadenylated RNAs with a length ranging from 200 to 100,000 nucleotides. Despite their lack of protein-coding capacity, lncRNAs have been increasingly recognised for their crucial roles in diverse cellular processes and functions. High-throughput RNA sequencing across various species led to the discovery and classification of lncRNAs and demonstrated that a large portion of lncRNAs exhibit lower abundance and limited conservation when compared to their coding counterparts.

The human genome may comprise approximately 170,000 lncRNAs; however, the function of many of them remains unknown [14,15]. Based on genome location, lncRNAs can be classified as intronic ncRNAs located in the introns of protein-coding genes, long intergenic ncRNAs (lincRNAs) that are situated between the protein-coding genes, enhancer-like ncRNAs (eRNAs), natural antisense transcripts (NATs), and transcribed ultra-conserved regions (T-UCRs) [16,17]. However, as the number of new lncRNAs is increasing, some of these molecules have failed to fit into specified groups; therefore, a new classification comprising eight broader categories has been suggested [18,19]. The proposed eight categories include intergenic, intronic, divergent, convergent, enhancer RNA, overlapping sense, overlapping antisense, as well as miRNA host genes. Still, this division is not final, as new data on these molecules’ functions and mechanisms are constantly gained.

The intricacies of the diverse mechanisms attributed to lncRNAs, coupled with their varying levels of expression, contribute to the complexity of the physiological roles of these noncoding RNAs [12,18,20]. LncRNAs, despite being considered in the past as non-functional byproducts of gene transcription, turned out to be crucial regulators of various physiological and pathological processes since they regulate the transcription and epigenetic chromatin remodelling [21,22,23,24]. LncRNAs are involved in every aspect of body functioning, as they act as molecular signals, scaffolds, decoys, enhancers, and guides [25,26,27,28,29]. These RNA molecules, serving as miRNA sponges, affect the stability of messenger RNAs (mRNAs) as well as the initiation of translation and, thus, they play an important role in the regulation of gene expression. LncRNAs influence gene expression at the transcriptional level by interacting with chromatin, modulating the activity of transcription factors, and regulating the accessibility of regulatory elements. Many lncRNAs have been implicated in the regulation of embryonic development and tissue differentiation. They can control the fate of cells during development and contribute to the establishment of cell identity. LncRNAs are involved in the regulation of the cell cycle by influencing key checkpoints and cell cycle progression. They can modulate the expression of genes involved in cell cycle control and contribute to cellular proliferation or differentiation.

Most intronic lncRNAs regulate either the transcription or alternative splicing of coding genes [25]. LncRNAs modulate the expression of neighbouring genes in cis-conformation, but also genes not closely located in trans-regulation [30]. Moreover, they were found to show cell-type-specific expression patterns and to be present in specific subcellular compartments [1]. The results of studies have revealed that subcellular localisation of lncRNAs determines their function [25]. In the cytoplasm, lncRNAs can modulate the translation of target gene mRNAs and the stability, as well as affect the expression of target genes via the regulation of distribution of miRNAs on their targets (competitive endogenous RNA). In turn, in the nucleus, lncRNAs act as co-activators/repressors, which reprogram gene expression, as well as molecular scaffolds. Moreover, they can act extracellularly through extracellular vesicle-derived lncRNAs. LncRNAs can also regulate gene expression after transcription by affecting mRNA splicing, stability, and translation. They may act as molecular decoys, competing for miRNA-binding sites or interacting with RNA-binding proteins. Furthermore, lncRNAs modulate post-translational modifications [21,31]. They can modify the chromatin structure, and affect the function of proteins, including transcription factors. LncRNAs can interact with proteins to form ribonucleoprotein complexes, influencing protein stability, localisation, and activity. These interactions may play a role in diverse cellular processes.

They are also involved in the epigenetic status of target genes [25]. LncRNAs participate in epigenetic regulation by influencing DNA methylation, histone modification, and chromatin remodelling. They can recruit chromatin-modifying complexes to specific genomic loci, thereby regulating the chromatin structure and gene expression. These molecules do not encode functional proteins; however, they can encode short peptides that modulate gene expression [32]. Some lncRNAs play a role in organising and maintaining the three-dimensional structure of the cell nucleus. They contribute to the formation of nuclear bodies and influence the spatial arrangement of genomic regions.

LncRNAs participate in the regulation of immune responses by modulating the expression of genes involved in immune cell development, activation, and function. They contribute to the fine-tuning of the immune system.

Various studies have demonstrated the alterations of many lncRNAs during development but also in disease-related processes. Certain lncRNAs are involved in cellular stress responses, including responses to oxidative stress, hypoxia, and other environmental challenges. They may help cells adapt to changing conditions and maintain homeostasis.

Dysregulation of lncRNA expression is often associated with various diseases, including cancer, cardiovascular diseases, neurodegenerative disorders, and metabolic disorders [33,34]. Some lncRNAs may function as oncogenes or tumour suppressors, influencing cancer development and progression. A few studies demonstrated the role of some lncRNAs in cholesterol metabolism and CVD [11,35,36]. Still, the mechanisms behind these effects are poorly understood.

This article focuses on the impact of various lncRNAs on lipoprotein metabolism.

## 3. High-Density Lipoproteins, Reverse Cholesterol Transport and Cardiovascular Risk

Cholesterol plays a crucial role as a fundamental building block of cell membranes and myelin sheaths [1]. Beyond its structural significance, cholesterol serves as a vital precursor in the biosynthesis of bile acids, vitamin D, and steroid hormones. Imbalances in cholesterol processing and metabolism have been linked to the development of cardiometabolic conditions, such as type 2 diabetes and atherosclerosis [37,38]. Considering that mammalian cells lack the ability to break down cholesterol, a physiological process called reverse cholesterol transport (RCT) becomes essential to remove excessive amounts. In the course of RCT, surplus cholesterol is transported from peripheral tissues to the liver, where it can be eliminated through excretion into the faeces [39,40]. HDL is the key lipoprotein involved in this process. It constitutes a large molecule with a complex structure, encompassing various lipids, proteins, and miRNAs [23]. HDL, along with its principal protein constituent—apolipoprotein AI (ApoAI), the primary sterol transporter—is involved in the transport of cholesterol from peripheral cells to the liver subsequent to cholesterol efflux mediated by ATP-binding cassette (ABC) transporters ABCG1 and ABCA1 [39,40]. ABCA1 emerges as a key player in cholesterol efflux from cells and contributes to the synthesis of HDL-C molecules, which play a primary role in transporting cholesterol back to the liver. Also, scavenger receptor class B type 1 (SR-B1), associated with HDL, participates in reverse cholesterol transport by promoting cholesterol efflux from macrophages, facilitating the uptake of HDL cholesterol by hepatocytes, and directing it toward bile [41]. Various transporters, including adenosine triphosphate (ATP)-binding cassette transporter B11 (ABCB11), ABCB4, ABCG5/G8, and ATPase class I type 8B member 1 (ATP8B1), are integral to the biliary secretion process [42].

RCT reduces circulating cholesterol levels, thereby diminishing the risk of CVD [43,44]. The results of some studies demonstrated that RCT-mediated cholesterol efflux from foam cells impeded the progression of atherosclerotic lesions and stimulated the regression of pre-existing plaques. HDL particles have been implicated in cardiovascular protection through their roles in the regulation of cholesterol efflux from peripheral tissues, anti-inflammatory and antioxidant activities, promotion of nitric oxide (NO) activity, and support for angiogenesis [45,46,47]. Epidemiological studies have observed an inverse association between HDL levels and cardiovascular (CV) risk; however, clinical trials that aimed at elevating HDL-C levels to mitigate cardiovascular events turned out to be failures. This highlights the importance of HDL’s biological activities or functions over its plasma levels in pathological conditions [23,48,49,50]. Moreover, genetic variant studies have not identified a positive correlation between plasma HDL levels and the development of coronary artery disease (CAD) [51]. Growing evidence suggests that normal HDL can transform into dysfunctional HDL particles in disease states, influencing vascular endothelial cell function in distinct ways [46,52,53].

Recent research in the cardiovascular field has brought to light the crucial roles of lncRNAs in cardiac development, pathophysiology, and lipid metabolism. These studies have unveiled the potential of lncRNAs as novel therapeutic targets as well as biomarkers for cardiovascular diseases [12].

## 4. LncRNAs Affecting Lipoproteins, Lipid Metabolism, and Atherosclerosis Risk

**Figure 1:**
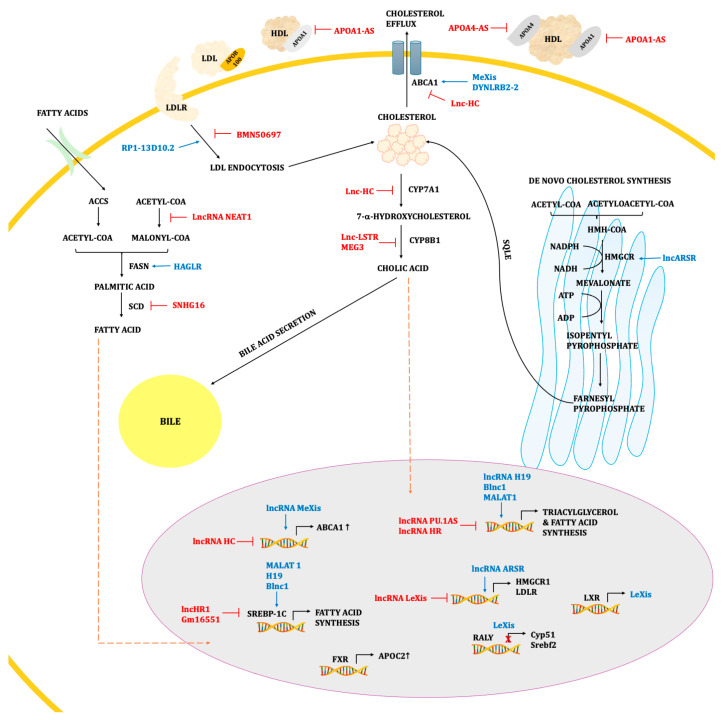
The summary of the possible roles of lncRNAs in lipid metabolism. Blue arrows indicate activation of pathway, red symbols indicate blocking of pathway; black arrows show the direction of pathway, while dotted yellow arrows show the downstream signalling. ATGL, adipose triglyceride lipase; CRP, C-reactive protein; CYP7a1, cholesterol 7α- hydroxylase; HMGCR, HMG-CoA reductase; LDL-C, low-density lipoprotein cholesterol; LDLR, low-density lipoprotein receptor; LPL, of lipoprotein lipase; NAFLD, nonalcoholic fatty liver disease; TG, triglyceride; VLDL-C, very-low-density lipoprotein cholesterol.

LncRNAs’ expression in cardiomyocytes and vascular endothelial cells has been suggested to play role in cardiovascular development and disease states [54]. These molecules play a pivotal role in the coordination of adipogenesis processes, as well as the metabolism and transport of fatty acids, cholesterol, and phospholipids, and the formation of HDLs and low-density lipoproteins (LDLs) (Figure 1) [24]. LncRNAs target key transcription factors involved in the regulation of lipid metabolism, such as peroxisome proliferator-activated receptor γ (PPARγ), liver X receptors (LXRs), and sterol regulatory element-binding proteins (SREBPs). LncRNAs have demonstrated the ability to modify the expression, localisation, or actions of transcription factors (e.g., SREBP and LXR). These factors modulate opposing gene pathways, supporting either cholesterol/fatty acid synthesis or cholesterol efflux/export. For instance, MALAT1 (metastasis-associated lung adenocarcinoma transcript 1) and lncRNA H19 stabilise the SREBP-1c protein, promoting hepatic lipogenesis [55]. In turn, lncHR1 blocks the SREBP-1c promoter, reducing hepatic and circulating triglyceride levels [56].

LncRNAs also play crucial roles in apoptosis and autophagy of vascular endothelial cells, smooth-muscle cell proliferation, foam cell formation, and lipid metabolism, all closely linked with atherosclerosis [21,57]. Altered expression of lncRNAs has the potential to influence specific mRNAs, leading to alterations in, e.g., HDL-C levels. LncRNAs also play a regulatory role in cholesterol and triglyceride metabolism [12]. Some studies have implied that lncRNAs are important regulators of HDL metabolism and HDL-induced alterations in vascular endothelial function, although the details remain unclear. It has been suggested that in patients with CVD and hypercholesterolaemia, dysfunctional HDL may trigger abnormal expression of lncRNAs in vascular endothelial cells, thereby adversely affecting vascular function [23,34]. The impact of lncRNAs related to HDL on endothelial function is due to the regulation of the expression of genes that serve as ultimate effectors. In the study of Liu et al. [23], the comparison of effects of HDL isolated from individuals with CVD and hypercholesterolaemia, with molecules from normal subjects, revealed that the first one contributed to the differential expression of transcripts in human umbilical vein endothelial cells (HUVECs). These transcripts were deemed crucial in the modulation of vascular endothelial function and related diseases. Bioinformatics analysis revealed intricate relationships and interactions among HDL-related lncRNAs, encoding genes, and miRNAs that orchestrated regulation of gene and protein expressions. This regulatory network may finally impact the function of vascular endothelial cells following the exposure to HDL. However, the study of lncRNAs in the cardiovascular system is still in its early stages [12].

Gaining a deeper understanding of the regulatory functions of lncRNAs in dyslipidaemia, atherosclerosis, and adipogenesis holds the potential to guide the development of effective strategies for treating these diseases [24]. Below, we summarise the sparse available information concerning the role of various lncRNAs in lipid metabolism disorders and atherosclerosis.

The comparison of genome-wide expression profiles of lncRNA and mRNA obtained from individuals with low HDL cholesterol disease and healthy counterparts, utilising microarray technology, unveiled a substantial number of differentially expressed lncRNAs and mRNAs [34]. Analyses based on Gene Ontology (GO) and the Kyoto Encyclopaedia of Genes and Genomes (KEGG) pathway disclosed the involvement of these differentially expressed genes in diverse cellular components, cellular processes, and molecular functions. The molecular mechanisms underlying the contribution of lncRNAs to low HDL-C disease involved associations between multiple lncRNAs and mRNAs. The co-expression network reported by Wang et al. [34] was suggested to pose a robust foundation for predicting the functions of lncRNAs. Pathway analysis led to the identification of 23 pathways associated with low HDL-C disease. Notably, platelet activation (KEGG ID: hsa04611) emerged as the most significantly enriched pathway, involving 14 differentially expressed genes. Through genomic annotation, enrichment analysis, co-expression analysis, and target gene prediction, 10 lncRNAs with accompanying target genes were identified. The combination of GO, the KEGG pathway, and disease enrichment analyses enabled the identification of co-expressed lncRNAs AC068234.2–202 and AP001033.3–201 and their target genes integrin subunit beta 3 *(ITGB3)* and thromboxane A2 receptor *(TBXA2R)* [34]. Differential expression of *ITGB3* and *TBXA2R* between two groups was associated with the platelet activation pathway and cardiovascular disease.

Presented below lncRNA has been divided into three groups, one containing lncRNAs that promote lipid disorders/atherosclerosis or CAD, the second with lncRNAs with beneficial effect and third one with not yet confirmed effect or dual actions.

### 4.1. LncRNA with Beneficial Effects

#### 4.1.1. NFIA Antisense RNA 1

NFIA antisense RNA 1 (NFIA-AS1/RP5-833A20.1) is another lncRNA that has been implicated in the modulation of cholesterol homeostasis, inflammatory responses, and foam cell formation through its interaction with miRNA, specifically miR-382-5p and subsequent inhibition of nuclear factor Ia (NFIA) [23]. *RP5-833A20.1* is situated within intron 2 of the *NFIA* gene. The result of a study demonstrated that lentivirus-mediated NFIA overexpression resulted in increased circulation of HDL-C, reduced levels of LDL-C, and very low density lipoprotein (VLDL-C), contributing to atherosclerosis regression in apolipoprotein E (apoE) knockout mice [58]. Thus, RP5-833A20.1 represents a potential therapeutic target for lipid-related diseases, offering avenues for ameliorating atherosclerosis [23]. Exposure to oxidized low-density lipoprotein or acetylated LDLs have been shown to enhance the expression of lncRNA RP5-833A20.1 in human THP-1 (human monocytic cell line derived from an acute monocytic leukaemia patient) macrophage-derived foam cells [25]. lncRNA RP5-833A20.1 in turn triggers hsa-miR-382-5p expression, leading to the upregulation of nuclear factor IA that acts as a major regulator involved in lipid homeostasis and adipocyte differentiation. Reduced nuclear factor IA negatively impacts reverse cholesterol transport and disrupts cholesterol homeostasis [58].

**Summary**:
**Name/organism:** RP5-833A20.1/ NFIA antisense RNA 1/NFIA-AS1; Homo sapiens
**Databases &Code:** Ensembl ENSG00000237853; HGNC: 40402; NCBI Gene: 645030
**Chromosomal location& size:** 1p31.3: 61,248,945–61,253,510 reverse strand; 4 exons, is associated with 1718 variant alleles
**Interaction with other molecules:** hsa-miR-382-5p
**Biological consequence of such interaction:** Increases circulation of HDL-C, reduces levels of LDL-C, and VLDL-C

#### 4.1.2. Liver-Expressed LXR-Induced Sequence

Liver X Receptor (LXR) plays a pivotal role in modulating genes associated with cholesterol homeostasis and contributes to the pathogenesis of cardiovascular diseases [59]. In mouse models subjected to a Western diet or pharmacological activation of LXR, the expression of the LXR-dependent hepatic lncRNA LeXis was found to be considerably enhanced, which translated into lower serum and hepatic cholesterol levels. Under conditions of excessive amounts of cholesterol, LXR triggers the expression of LeXis in the liver, which, in turn, mitigates cholesterol synthesis by impeding the recruitment of RNA polymerase II to Srebf2 and its transcriptional targets [12,60]. The LXR-regulated transcript, LeXis, is situated proximal to the *Abca1* locus in mouse hepatocytes [60]. Hepatic LeXis strongly affects the expression of genes related to cholesterol biosynthesis and hepatic cholesterol content. Mice lacking *LeXis* exhibit increased expression of cholesterol biosynthetic enzymes and greater hepatic cholesterol content. In turn, the overexpression of LeXis markedly suppresses the expression of hepatic cholesterogenic genes. Raly, a heterogeneous ribonucleoprotein formerly linked to sterol regulatory element-binding protein 2 (SREBP-2), has been identified as a novel binding partner for LeXis. The regulatory impact of LeXis on the expression of cholesterol biosynthetic genes seems to be mediated by Raly, as its ability to control hepatic cholesterol metabolism is compromised upon Raly knockdown. The experiment with adenovirus-mediated knockdown of Raly in mouse liver revealed diminished serum cholesterol, mimicking the effect of LeXis expression. Moreover, LeXis activity in vivo was demonstrated to be dependent on Raly, since the ability of LeXis to modify hepatic gene expression and affect serum cholesterol levels was impaired in the absence of Raly. Moreover, in mice with a deleted *SREBP2* regulator, LeXis could not decrease cholesterol levels, indicating a role for the SREBP2 pathway in the function of LeXis [60].

**Summary**:
**Name/organism:** LeXis/CT70 (cancer/testis associated transcript 70); Homo sapiens
**Databases &Code:** Ensembl ENSG00000230013; HGNC:37195
**Chromosomal location& size:** 9q31.1
**Interaction with other molecules:** Raly
**Biological consequence of such interaction:** Modulates expression of cholesterol biosynthetic genes

#### 4.1.3. Macrophage-Expressed LXR-Induced Sequence

Macrophage-expressed LXR-induced sequence (MeXis) is located in the nucleus and exercises control over chromosome architecture within the *Abca1* locus [1]. Direct interaction with DDX17 is a key aspect of MeXis functionality. The MeXis level increases substantially upon the activation of LXRs in macrophages [1,61]. As aforementioned, LXRs are sterol-activated nuclear receptors that modulate the expression of genes involved in reverse cholesterol transport. LXR-mediated upregulation of ABCA1 expression is crucial for cholesterol efflux. Higher MeXis levels subsequently amplify Abca1 expression, enhancing macrophage cholesterol efflux. In the mouse liver, MeXis was demonstrated to serve as a process amplifier since it interacted with and guided the transcriptional coactivator DDX17 (DEAD-box Helicase 17) to bind to the ABCA1 promoter [62]. MeXis plays a significant role in lipid metabolism by regulating ABCA1, a pivotal factor in the control of cholesterol efflux and the formation of HDL [21]. Stable overexpression of MeXis in macrophages boosts ABCA1 expression, leading to more effective cholesterol elimination and considerably decreasing the likelihood of atherosclerosis. Mice lacking the *MeXis* gene were found to have reduced tissue-selective expression of ABCA1, resulting in a two-fold increase in the probability of vascular occlusion compared to normal mice [61]. Significantly diminished ABCA1 expression in macrophages translated into exacerbated progression of atherosclerosis. A similar transcript (TCONS00016111) located proximal to the *ABCA1* locus in humans exhibits a comparable impact, influencing ABCA1 levels and cholesterol efflux in a human macrophage cell line (THP-1). These observations align with the significant association between a single-nucleotide polymorphism (SNP) overlapping the TCONS00016111 transcript and coronary disease, implying that the LXR–MeXis–ABCA1 axis may play a role in the regulation of macrophage sterol metabolism in humans. Considering that the use of LXR activators is associated in vivo with adverse effects resulting from the stimulation of hepatic fatty acid synthesis and steatosis, the identification of MeXis that serve as a specific regulator of LXR-induced ABCA1 expression in macrophages offers a novel opportunity to promote RCT in vivo without causing detrimental effects. Therefore, targeting the LXR/MeXis/ABCA1 pathway presents a promising strategy for treating or preventing atherosclerotic disease by modulating cellular cholesterol transport.

**Summary**:
**Name/organism:** MeXis/AI427809/LOC381524; Homo sapiens
**Databases &Code:** Ensembl ENSMUSG00000086712.3
**Chromosomal location& size:** Chromosome 4: 53,261,356–53,270,232 reverse strand.
4 transcripts (splice variants), 3 orthologues and is associated with 4 phenotypes
**Interaction with other molecules:** Raly, DDX17
**Biological consequence of such interaction:** Macrophage expressed LXRa (NR1H3)-dependent amplifier of Abca1 transcription lncRNA

#### 4.1.4. LncRNA RP1-13D10.2

The clearance of LDL particles within the bloodstream requires interactions with low-density lipoprotein receptors [24]. LncRNA RP1-13D10.2 has been identified as a molecule that can promote the increase in LDL receptors’ expression, thereby enhancing the internalisation of plasma LDL-C in human Huh7 and HepG2 hepatocyte cell lines. Apart from LDLR transcript levels, overexpression of RP1-13D10.2 enhanced LDL uptake, and decreased media levels of apolipoprotein B [63]. The expression levels of RP1-13D10.2 appear to be sterol-regulated. Moreover, Zelcer et al. [64] demonstrated increased RP1-13D10.2 expression levels after incubation with an LXR agonist, which implies that it may also be an LXR target gene. Mitchel et al. [64] observed an association between inter-individual variation in the magnitude of sterol regulation with statin-induced changes in LDLC from a panel of immortalised lymphoblastoid cell lines obtained from participants of a statin clinical trial. The elevation in RP1-13D10.2 levels was observed in individuals displaying a high LDL-C response to statin therapy, while those with a diminished LDL-C response exhibited decreased RP1-13D10.2 levels. This finding suggests that RP1-13D10.2 may be a novel lipid regulator and could play a pivotal role in statin-induced reduction of LDL-C levels [64]. Therefore, it could serve as biomarker, enabling the estimation of statin efficacy for plasma LDLC lowering.

**Summary**:
**Name/organism:** RP1-13D10.2;
**Interaction with other molecules:** LXR, SREBF2
**Biological consequence of such interaction:** Regulates LDLR gene expression in a sterol-responsive and SNP genotype–dependent manner in vitro

#### 4.1.5. LncLSTR

The liver-enriched long non-coding RNA is an intergenic lncRNA located in a region of the mouse genome that is syntenic to human chromosome 1q25. Clear human homologues have still not been identified using the BLAST algorithm [33]. LncLSTR expression appears to be regulated in response to metabolic states [33]. During fasting, the expression of LncLSTR is diminished, while it shows a substantial upregulation upon re-feeding. However, the precise molecular mechanisms behind its expression related to metabolic adaptation remain unidentified. The use of small hairpin RNAs (shRNA) adenovirus, which inhibits the expression of LncLSTR to elucidate the specific role of LncLSTR in the regulation of lipid metabolism, revealed a consequent considerable decrease in circulating triglycerides (TAGs) in both wild-type (WT) and *Apoe−/−* mice. LncLSTR was demonstrated to markedly enhance the expression of apolipoprotein C2 (ApoC2), which acts as a positive regulator of lipoprotein lipase (LPL). LPL enzyme is involved in the catabolism of triglyceride-rich lipoproteins (TRLs) and facilitates the uptake and storage of fatty acids in oxidative tissues. Li et al. [33] demonstrated that restoring normal ApoC2 expression through liver-specific ApoC2 knockdown reverses the reduced plasma TG levels associated with lncLSTR knockdown.

The involvement of lncLSTR in systemic triglyceride metabolism implies the potential utilisation of these molecules by organisms to orchestrate metabolic processes across various organs. The modulation of TDP-43/Cyp8b1 by lncLSTR subsequently influences the FXR/ApoC2 pathway, thereby regulating triglyceride clearance. This underscores the intricate connections between lncRNAs and established signalling and metabolic networks. FXR activity affects diverse metabolic pathways, encompassing bile acid synthesis and lipid homeostasis [65], with activation of this pathway yielding pleiotropic beneficial effects in the context of metabolic disorders [66]. Moreover, TDP-43, apart from acting as a transcriptional repressor, serves as a key regulator of microRNA and mRNA processing [67]. Although FXR deficit does not fully hamper the lipid-lowering effects of lncLSTR depletion, the interaction between lncLSTR and TAR DNA-binding protein 43 (TDP-43) may involve additional molecular and physiological functions, warranting further exploration in future investigations.

**Summary**:
**Name/organism:** LncLSTR (lncRNA liver-specific triglyceride regulator);
**Chromosomal location& size:** Syntenic to human chromosome 1q25
**Interaction with other molecules:** TDP-43
**Biological consequence of such interaction:** Modulates bile acid composition to regulate APOC2 expression, via FXR,85 and to control serum triglyceride levels

#### 4.1.6. Cholesterol-Induced Regulator of Metabolism RNA 

CHROME (Cholesterol-induced regulator of metabolism RNA), also known as PRKRA-AS1, is a primate-specific lncRNA situated on human chromosome 2q31.2 [68]. This lncRNA is intricately associated with cellular and systemic cholesterol homeostasis [25]. The upregulation of this lncRNA has been observed in the plasma and arterial plaques of patients with CVD. CHROME expression was suggested to be modified by nutritional and cellular cholesterol levels through sterol-activated LXR transcription factors [69]. Functionally, CHROME promotes cholesterol secretion and HDL synthesis via suppressing the activity of specific miRNAs [69]. Experimental interventions, including gain- and loss-of-function approaches, demonstrated that CHROME could simultaneously inhibit the actions of functionally related miRNAs, such as miR-27b, miR-33a/b, and miR-128 [70]. In turn, the silencing of CHROME in human hepatocytes and macrophages results in increased expression of miR-27b, miR-33a, miR-33b, and miR-128, consequently reducing the levels of their common target genes, especially ABCA1, a key regulator of de novo HDL synthesis [70]. Therefore, the knockdown of CHROME disrupted cholesterol efflux and impaired the formation of nascent high-density lipoprotein. Thus, CHROME emerges as a potential clinical biomarker for the treatment of cholesterol-related diseases.

**Summary**:
**Name/organism:** CHROME, PRKRA-AS1; Homo sapiens
**Databases &Code:** Ensemble: ENSG00000223960.9; HGNC:54059
**Chromosomal location& size:** 2q31.2: 178,413,635–178,440,243 forward strand; 35 transcripts (splice variants)
**Interaction with other molecules:** miR-27b, miR-33a, miR-33b, and miR-128
**Biological consequence of such interaction:** Promotes cholesterol secretion and HDL synthesis via suppressing the activity of specific miRNAs

#### 4.1.7. Lipid-Droplet Transporter 

Lipid-droplet transporter (LIPTER) has been demonstrated to aid in the transport of lipid droplets (LDs) within human cardiomyocytes [71]. LIPTER deficiency has been found to impair LD transport and metabolism, leading to compromised mitochondrial function and cardiomyocytes’ viability. LDs are essential cellular components crucial for maintaining lipid balance within cells. Their accumulation within myocytes has been linked to heart diseases associated with metabolic disorders. Lipid droplets play pivotal roles in intracellular lipid transport, lipid balance, and membrane synthesis [72,73]. In conditions of elevated lipid levels, LDs accumulate in myocardium and cardiomyocytes, leading to a condition known as cardiac steatosis [73]. Initially, higher LD levels provide temporary protection to the heart by storing excess fatty acids (FAs) in cardiomyocytes; however, prolonged lipid accumulation can lead to harmful consequences, such as cell death, tissue damage, and impaired cardiac function (lipotoxicity) [74,75]. Moreover, LDs also play a crucial role in cellular metabolism, particularly during nutrient deprivation, since at that time they are moved to mitochondria for energy production through β-oxidation [72]. Han et al. [71] reported that LIPTER might function as an RNA linker that enables LD transport in cardiomyocytes through linking LD and cytoskeleton via binding phosphatidic acid (PA)/phosphatidylinositol 4-phosphate (PI4P) and myosin heavy chain 10 (MYH10) motor protein, respectively. LIPTER interacts with phospholipids on LD membranes, such as phosphatidic acid (PA) and phosphatidylinositol 4-phosphate (PI4P), as well as with the MYH10 motor protein, facilitating the connection between LDs and the cytoskeleton for intracellular transport. LIPTER overexpression has been shown to alleviate cardiomyopathies and preserve cardiac function in mouse models of obesity and diabetes, suggesting its potential clinical significance in the treatment of heart diseases associated with metabolic syndromes.

**Summary**:
**Name/organism:** LIPTER/LINC00881; Homo sapiens
**Databases &Code:** Ensemble: ENSG00000241135.8; HGNC: 48567; NCBI Gene: 100498859
**Chromosomal location& size:** 3q25.31: 157,089,634–157,135,557 forward strand; 11 transcripts
**Interaction with other molecules:** Phosphatidic acid, phosphatidylin-ositol 4-phosphate MYH10 motor protein
**Biological consequence of such interaction:** Facilitates the connection between LDs and the cytoskeleton for intracellular transport

#### 4.1.8. Regulator of Hyperlipidaemia Long Noncoding RNA

Recently, regulator of hyperlipidaemia long noncoding RNA (lncRHPL) has been found to regulate hepatic VLDL secretion by modulating the hnRNPU/BMAL1/MTTP axis [76]. Its expression was notably reduced in mice subjected to a high-fat diet (HFD) and in primary hepatocytes treated with oleic acid. Functional studies revealed that lncRHL suppressed hepatic VLDL secretion without impacting lipogenesis or fatty acid oxidation. Moreover, lncRHL expression was reported to be regulated by nutritional signalling and to take part in the pathogenesis of dyslipidaemia and non-alcoholic fatty liver disease (NAFLD). Further insights into lncRHL’s mechanism unveiled its direct interaction with hnRNPU, which is an RNA-binding protein crucial for mRNA stabilisation and regulation of downstream molecules [77]. LncRHL depletion led to reduced levels of hnRNPU protein via proteasome-mediated ubiquitination, while lncRHL overexpression stabilised hnRNPU levels [78]. Shen et al. [78] also demonstrated that lncRHL knockdown resulted in reduced BMAL1 expression, which in turn influenced VLDL secretion and altered the expression of BMAL1’s downstream targets (*Gata4*, *Abcg5*, and *Abcg8*). In turn, restoration of BMAL1 promoted VLDL secretion and expression of downstream targets in lncRHL knockdown primary hepatocytes [78]. Therefore, it appears that BMAL1 serves as the downstream target of lncRHL in the modulation of hepatic VLDL secretion. Overall, lncRHL regulates hepatic VLDL-TAG by regulating the hnRNPU/BMAL1/MTTP axis. Significant downregulation of lncRHL expression in the high-fat diet suggests its pivotal role in HFD-induced hyperlipidaemia. Diminished levels of lncRHL in the liver could pose a potential risk factor for CVD.

**Summary**:
**Name/organism:** lncRHPL; Mus musculus (house mouse)
**Databases &Code:** NIH Gene ID: 105244982
**Chromosomal location& size:** Chromosome 8
**Interaction with other molecules:** hnRNPU, BMAL1
**Biological consequence of such interaction:** Modulates hepatic VLDL secretion.

#### 4.1.9. LncNONMUG027912

LncNONMMUG027912 (referred to as lnc027912) was found to be involved in cholesterol metabolism [79]. Chu et al. [80] demonstrated that increased expression of lnc027912 in the murine hepatocyte cell line AML12 treated with oleic acid (OA) and palmitic acid led to a marked reduction in lipid accumulation, TG levels, and expression of lipid biosynthesis genes. Mechanistically, lnc027912 upregulated the expression of phosphorylated AMP-activated protein kinase α (p-AMPKα), reduced phosphorylated mammalian target of rapamycin (p-mTOR) levels, suppressed nuclear expression of sterol regulatory element-binding protein 1C (SREBP1C), attenuated the promoter activity of SREBP1C, and hindered the expression of lipid synthesis genes. The authors demonstrated that lnc027912 decreased lipid accumulation and liver inflammation via the AMPKα/mTOR signalling axis in a NAFLD mouse model.

**Summary**:
**Name/organism:** LncNONMMUG027912/lnc027912
**Interaction with other molecules:** AMPKα/mTOR signalling axis
**Biological consequence of such interaction:** Upregulates p-AMPKα, reduces p-mTOR levels, suppresses nuclear expression of SREBP1C, and hinders the expression of lipid synthesis genes

#### 4.1.10. Maternally Expressed 3

Maternally Expressed Gene 3 (*MEG3*), situated on chromosome 14q32.2, is believed to be implicated in human lipid metabolic disorders [68]. A recent study reported a reduction in MEG3 expression in serum samples obtained from individuals with atherosclerosis [80]. MEG3 deficiency significantly mitigated hepatic TG accumulation in both HFD mice and obese (ob/ob) mice [81,82]. Furthermore, MEG3 played a beneficial role in alleviating NAFLD in a mouse model [81]. In mice with NAFLD, MEG3 overexpression showed a negative correlation with various lipogenesis-related genes, such as SREBP-1, carbohydrate response element-binding protein (ChREBP), LXRα, acetyl-CoA carboxylase 1 (ACC1), stearyl-coenzyme A desaturase 1 (SCD1), and fatty acid synthase (FAS) [83]. The results of bioinformatic analysis and mechanistic studies demonstrated that MEG3 competitively bound to miR-21 with LRP6, subsequently repressing the mTOR pathway and hepatic lipogenesis [83].

**Summary**:
**Name/organism:** MEG3/GTL2/LINC00023/NCRNA00023/ONCO-LNCRNA-83;
**Databases &Code:** ENSG00000214548.18; HGNC (14575); NCBI Gene (55384); OMIM^®^ (605636); Open Targets Plat-form (ENSG00000214548)
**Chromosomal location& size:** Chromosome 14: 100,779,410–100,861,031 forward strand;
50 transcripts and is associated with 6 phenotypes
**Interaction with other molecules:** miR-21
**Biological consequence of such interaction:** Modulates hepatic lipogenesis

#### 4.1.11. Cyclin-Dependent Kinase Inhibitor 2B Antisense RNA 1

Also, lncRNA ANRIL (cyclin-dependent kinase inhibitor 2B antisense RNA 1; CDKN2B-AS1) was found to play important roles in various diseases, including atherosclerosis, diabetes, and cancer [84]. According to studies, ANRIL expression is frequently downregulated in atherosclerotic plaque tissue and THP-1 macrophage-derived foam cells. Moreover, overexpression of this lncRNA was found to enhance cholesterol efflux by suppressing the expression of a disintegrin and metalloprotease 10 domain (ADAM10) in atherosclerosis. The recruitment of DNA methyltransferase 1 (DNMT1) to the promoter region of ADAM10 is associated with initiation of the methylation [84]. In turn, the increased ADAM10 expression led to heightened intracellular cholesterol accumulation.

**Summary**:
**Name/organism:** ANRIL/CDKN2B-AS1/RP11-145E5.4/NCRNA00089/p15AS/PCAT12; Homo sapiens
**Databases &Code:** HGNC: 34341; Ensembl: ENSG00000240498; NCBI Gene: 100048912; OMIM^®^: 613149
**Chromosomal location& size:** located within the CDKN2B-CDKN2A gene cluster at chromosome 9p21.3: 21,994,139–22,128,103 forward strand; 28 transcripts
**Interaction with other molecules:** polycomb repressive complex-1 (PRC1) and -2 (PRC2),
**Biological consequence of such interaction:** Epigenetic silencing of other genes in this cluster.

#### 4.1.12. HOXC Cluster Antisense RNA 1 

HOXC cluster antisense RNA 1 (HOXC-AS1) is situated in the chromosome 12q13.13. Microarray analysis and RT-PCR revealed decreased expression levels of both HOXC-AS1 and homeobox C6 (HOXC6) in human atherosclerotic plaques compared to normal intima tissues [85]. In turn, lentivirus-mediated overexpression of HOXC-AS1 limited oxidized LDL (Ox-LDL)-induced cholesterol accumulation by enhancing HOXC6 expression in THP-1 macrophages [85]. Various studies have suggested the involvement of HOX gene networks in human adipogenesis, with HOXC6 inhibiting intracellular lipid accumulation [76]. Therefore, it appears that HOXC-AS1 emerges as a promising therapeutic target for preventing atherosclerosis.

**Summary**:
**Name/organism:** HOXC-AS1/NONHSAG011268.2/HSALNG0091321; Homo sapiens
**Databases &Code:** HGNC: 43749; NCBI Gene: 100874363; Ensembl: ENSG00000250451
**Chromosomal location& size:** 12q13.13; Ch 12: 53,999,022–54,000,010 reverse strand; 2 transcripts
**Biological consequence of such interaction:** Inhibition of intracellular lipid accumulation

### 4.2. LncRNA with Adverse Effects

#### 4.2.1. AC068234.2–202 and AP001033.3–201

The lncRNA transcript AP001033.3–201, a product of the gene *AP001033.3*, consists of 3 exons and also lncRNA AC068234.2–202, an antisense transcript to ITGB3 and a product of the gene *AC068234*.2, comprises 3 exons. According to existing literature, ITGB3, as a key member of the integrin family, is associated with platelet aggregation, thrombus formation, atypical platelet activation and thrombosis, as well as inflammatory responses and atherosclerosis [86,87]. Similarly, TBXA2R is implicated in platelet function and aggregation, venous thrombosis, and the atherosclerotic process [88]. Alterations in TBXA2R distribution have been observed in various cardiovascular diseases, contributing to pathophysiological processes [34]. Based on their own research, Wang et al. [34] suggested that lncRNA AC068234.2–202 acts as a cis-regulator of ITGB3, while lncRNA AP001033.3–201 acts as a trans-regulator of TBXA2R. These regulatory actions contribute to the stimulation of platelet activation and, consequently, the pathogenesis of CVD. These findings lay the groundwork for further exploration of lncRNA functions, signalling pathways, and their roles in the context of low HDL-C disease.

**Summary**:
**Name/organism:** AC068234.2–202; Homo sapiens
**Databases &Code:** AC068234.2
**Chromosomal location& size:** Ch17:47,303,474–47,323,613 reverse strand; transcript with 3 exons, associated with 4518 variant alleles
**Interaction with other molecules:** TBXA2R
**Biological consequence of such interaction:** Possibly contribute to the trans-regulation of the protein-coding gene thromboxane A2 receptor (TBXA2R)
**Name/organism:** AP001033.3–201; Homo sapiens
**Databases &Code:** AP001033.3
**Chromosomal location& size:** Ch18: 9,310,522–9,334,445 reverse strand; transcript with 3 exons and 5282 reported variant alleles
**Interaction with other molecules:** antisense to ITGB3
**Biological consequence of such interaction:** Acts a cis-regulator of the protein-coding gene integrin subunit beta 3 (ITGB3)

#### 4.2.2. LncRNA ENST00000602558.1

This long non-coding RNA has been found to be differentially expressed between individuals with CAD and healthy controls. This lncRNA is located on chromosome 12q24.31, within susceptibility loci associated with triglycerides (TG), CVD, and HDL [89]. Similar to most lncRNA, its specific role and underlying mechanisms behind lipid alterations and atherosclerosis pathogenesis remain unclear. Notably, overexpression of ENST00000602558.1 was found to downregulate both ABCG1 mRNA and diminish protein levels, whereas the knockdown of *ENST00000602558.1* resulted in the upregulation of ABCG1 mRNA and enhanced protein expression [89]. Furthermore, Cai et al. [89] demonstrated that ENST00000602558.1 overexpression led to a significant reduction (by 30.38%, *p* < 0.001) in ABCG1-mediated cholesterol efflux to HDL from vascular smooth-muscle cells (VSMCs), while knockdown of ENST00000602558.1 enhanced ABCG1-mediated cholesterol efflux by 30.41% (*p* = 0.001) [89]. Apart from its impact on cholesterol efflux, ENST00000602558.1 overexpression promoted lipid accumulation and increased total cholesterol/triglyceride (TC/TG) levels. In turn, the knockdown of ENST00000602558.1 resulted in limited lipid accumulation and lower TC/TG levels in VSMCs. Importantly, ENST00000602558.1 was demonstrated to regulate ABCG1 expression and ABCG1-mediated cholesterol efflux in VSMCs via interacting with p65.

**Summary**:
**Name/organism:** ENST00000602558.1; Homo sapiens
**Databases &Code:** Ensembl: ENST00000602558.1
**Chromosomal location& size:** Chromosome 12: 123,971,457-123,971,714 reverse strand;
Exons: 1, Coding exons: 0, Transcript length: 258 bps; sense intronic to CCDC92
**Interaction with other molecules:** p65
**Biological consequence of such interaction:** Downregulates ABCG1 mRNA

#### 4.2.3. Long Intergenic Non-Protein Coding RNA 1228

Long intergenic non-protein coding RNA 1228 (lincRNA-DYNLRB2-2) expression in human macrophages is induced by ox-LDL [79]. This transcript plays a pivotal role in augmenting ABCA1-related cholesterol efflux and mitigating inflammatory responses through GPR119 in macrophage-derived foam cells [58]. The upregulation of DYNLRB2 expression in macrophage foam cells leads to an increased expression of ABCA1 and the G-protein-coupled receptor 119 (GPR119), thereby facilitating cholesterol efflux and diminishing neutral lipid accumulation. The precise involvement of DYNLRB2 in the regulation of lipoprotein metabolism and atherogenesis remains unclear. However, deprivation of DYNLRB2 has been associated with decreased circulating lipids and mitigated atherosclerosis. This implies that DYNLRB2 may exert an influence on lipoprotein metabolism and the progression of atherosclerosis.

**Summary**:
**Name/organism:** LINCRNA-DYNLRB2-2/LINC01228;
**Databases &Code:** Ensembl: ENST00000567966.1
**Chromosomal location& size:** Chromosome 16: 79,798,050–79,827,150 reverse strand; Size: 623 bp
**Interaction with other molecules:** GPR119
**Biological consequence of such interaction:** Facilitates cholesterol efflux and diminishes neutral lipid accumulation

#### 4.2.4. Taurine Upregulated Gene 1

LncRNA TUG1 (taurine upregulated gene 1) has emerged as a potential epigenetic modulator in diabetes and diabetes-related diseases [90]. Moreover, its involvement in fundamental metabolic processes, such as glucose homeostasis, insulin signalling, and lipid metabolism, has been demonstrated. TUG1 is extensively linked with several cholesterol efflux genes, including apolipoprotein M (ApoM), ABCA1, and ABCG1, and consequently with atherosclerosis. Elevated levels of TUG1 are associated with decreased expression of the aforementioned genes, consequently reducing the rate of cholesterol efflux. In turn, silencing TUG1 expression inhibited hyperlipidaemia and reduced atherosclerotic lesions in *ApoE−/−* mice subjected to a high-fat diet. Mechanistically, TUG1 competes with fragile X mental retardation syndrome-related protein 1 (FXR1), a negative regulator of ApoM, for binding to miR-92a [91]. TUG1 acts also as a sponge for miR-133a, thereby suppressing its ability to activate FGF1 (fibroblast growth factor 1). Consequent restoration of FGF1 counteracts the effects of miR-133a on cell proliferation, secretion of inflammatory factors, and apoptosis in macrophages treated with Ox-LDL [92]. Strategies aimed at modulating TUG1 expression levels or its interacting partners could potentially restore normal gene expression patterns, improve insulin sensitivity, enhance insulin secretion, and alleviate the progression of diabetic complications [90]. TUG.1 emerges as a potential novel biomarker for diagnosing atherosclerosis [68].

**Summary**:
**Name/organism:** TUG1/FLJ20618/LINC00080/NCRNA00080; Homo sapiens
**Databases &Code:** Ensembl: ENSG00000253352.10
**Chromosomal location& size:** 22q12.2: 30,969,245–30,979,395 forward strand; 20 transcripts (splice variants) and 9 orthologues
**Interaction with other molecules:** miR-92a, miR-133a
**Biological consequence of such interaction:** Suppression of FGF1activation

#### 4.2.5. Myocardial Infarction-Associated Transcript 

Long non-coding myocardial-infarction-associated transcript (MIAT) is a hypoxia-response gene located in the chromosome 22q12.1 region [68]. This lncRNA is found in heart and foetal brain tissues [93]. MIAT levels were significantly higher in the serum of patients with vulnerable atherosclerotic plaques [94]. In *ApoE−/−* mice, MIAT raised blood lipid levels, stimulated the formation of atherosclerotic plaques, increased lipid content, and reduced collagen content in the plaques [95]. The activity of MIAT in the formation of vulnerable plaques as well as atherosclerotic plaque progression in rats is mediated by the activation of the phosphatidylinositol 3-kinase (PI3K)/protein kinase B (Akt) PI3K/Akt signalling pathway. In turn, MIAT silencing hampered the progression of atherosclerosis in a mouse model of advanced atherosclerosis [94]. MIAT activation was also demonstrated to enhance angiogenesis and increase the expression of inflammatory factors (IL-1β, IL-6, and TNF-α) in mice with atherosclerosis [95]. MIAT was found to be the target of m6A modification. m6A demethylation and ox-LDL-induced AlkB homologue 1 (ALKBH1) enhanced MIAT activity through the hypoxia-inducible factor 1α (HIF1α) motif [96]. Deficiency of ALKBH1 or HIF1α significantly increased MIAT expression and m6A levels in vitro [97]. Additionally, MIAT has been demonstrated to affect various cellular functions, e.g., apoptosis [98]. Overexpression of MIAT can lead to microvascular dysfunction due to enhanced proliferation and migration of endothelial cells [99].

**Summary**:
**Name/organism:** MIAT/RNCR2/GOMAFU/C22orf35/LINC00066/NCRNA00066/lncRNA-MIAT; Homo sapiens
**Databases &Code:** HGNC: 33425; NCBI Gene: 440823; Ensembl: ENSG00000225783; OMIM^®^: 611082
**Chromosomal location& size:** 22q12.1: 26,646,411–26,676,475 forward strand; 30 transcripts (splice variants) and is associated with 1 phenotype
**Interaction with other molecules:** PI3K/Akt signalling pathway
**Biological consequence of such interaction:** May constitute a component of the nuclear matrix; enhances angiogenesis and increases the expression of inflammatory factors

#### 4.2.6. LncRNA RP11-728F11

LncRNA RP11-728F11.4 has been associated with CD36 levels in human monocyte-derived macrophages [25]. Dong et al. [100] revealed that RP11-728F11.4 alleviates the repressive activity of Ewing Sarcoma breakpoint region 1/EWS RNA binding protein 1 (EWSR1) on FXYD domain-containing ion transport regulator 6 (FXYD6) by attaching to its RNA recognition domain. This interaction results in elevated FXYD6 and, subsequently, it stimulates CD36 expression, which leads to intracellular cholesterol accumulation [100]. lncRNA RP11-728F11.4 contributes to the development of atherosclerosis by increasing the expression of FXYD6, which leads to intracellular cholesterol buildup and the production of proinflammatory cytokines [100].

**Summary**:
**Name/organism:** LncRNA RP11-728F11;
**Interaction with other molecules:** EWSR1 (Ewings sarcoma RNA binding protein-1)
**Biological consequence of such interaction:** Induction of cholesterol uptake in monocytes-derived macrophages and proinflammatory cytokine production

#### 4.2.7. lncRNA ENST00000416361

Knockdown of ENST00000416361 significantly reduced the levels of the inflammatory factors IL-6 and TNF-α, which are closely linked to CAD progression [101]. Moreover, it also led to the downregulation of SREBP1 and SREBP2, which are important in lipid synthesis. These two factors have been found to be significantly upregulated in CAD plasma samples. SREBP1, mainly found in the liver and adrenal gland, is involved in fatty acid metabolism, while SREBP2 participates in cholesterol biosynthesis and metabolism [102,103]. SREBPs’ upregulation was reported in CAD patients, and they pose risk factors, indicating CAD severity and poor lipid control [104]. LncRNA ENST00000416361 shows potential as a biomarker for CAD screening and diagnosis. The association of ENST00000416361 with inflammatory cytokines (IL-6 and TNF-α) and lipid metabolism-related genes (SREBP1 and SREBP2) suggests its involvement in atherosclerosis pathogenesis through inflammation and lipid metabolism.

**Summary**:
**Name/organism:** ENST00000416361; Homo sapiens
**Databases &Code:** Ensembl: ENST00000416361
**Chromosomal location& size:** 2102 bp
**Interaction with other molecules:** SREBP
**Biological consequence of such interaction:** Affects the occurrence and development of CAD

#### 4.2.8. LncRNA RAPIA

High expression of LncRNA RAPIA (associated with the progression and intervention of atherosclerosis) has been observed in the late stages of atherosclerosis and in macrophages [105]. In vivo, the blockage of RAPIA suppresses atherosclerosis progression and elicits atheroprotective effects comparable to those of atorvastatin on advanced plaques. This discovery underscores the potential role of noncoding RNAs in cardiovascular diseases, including atherosclerosis, and suggests that modulating their activity could represent a novel therapeutic approach for cardiovascular disease. RAPIA orchestrates the proliferation and apoptosis of macrophages via the miR-183-5p/ITGB1 (integrin β1) pathway. ITGB1 has been demonstrated to promote atherosclerosis progression [106]. In advanced atherosclerosis, RAPIA exhibits significant upregulation compared to both normal and early atherosclerosis groups, with this increase attributed to multiple factors. RAPIA expression may be facilitated by the transcription factor FoxO1 (forkhead box O1) [105].

**Summary**:
**Name/organism:** RAPIA;
**Chromosomal location& size:** 10,252 nucleotides
**Interaction with other molecules:** miR-183-5p-ItgB1 (integrin β1)
**Biological consequence of such interaction:** Coordination of proliferation and apoptosis of macrophages

#### 4.2.9. Nuclear Paraspeckle Assembly Transcript 1 

The nuclear paraspeckle assembly transcript 1 (NEAT1) is a lncRNA with a length of over 200 nucleotides that is crucial for regulating lipid droplet aggregation [107]. NEAT1 is produced by RNA polymerase II from the multiple endocrine neoplasias (MEN) type I region located on human chromosome 11q13 [108]. NEAT1 may play a significant role in atherosclerosis development, as blocking NEAT1 reduces lipid uptake in human macrophage THP-1 cells [109]. It is also implicated in myocardial ischemia–reperfusion injury by activating the MAPK pathway. Knockout of NEAT1 inhibits the release of inflammatory factors TNF-α, IL-1β, and IL-6, as well as production of matrix metalloproteinases enhanced by inflammation [110,111]. In human macrophages, silencing of NEAT1 boosts miR-342-3p expression and reduces the inflammatory response [109]. In turn, NEAT1 overexpression in myocardial cells blocks miR-495-3p, increases TNF-α, IL-1β, and IL-18 levels, and exacerbates ischemia–reperfusion injury [112]. NEAT1 regulates cardiovascular disease through inflammation. Chen et al. [107] demonstrated that NEAT1 levels in serum exosomes of patients with ST-elevation myocardial infarction (STEMI) were higher than those in the control group. In turn, Fan et al. [113] suggested that in hepatocellular carcinoma, NEAT1 influenced TG metabolism by upregulating miR-372-3p in RAPA-treated mice and cell models. Other studies showed that NEAT1 modulated adipose triglyceride lipase (ATGL) expression, with NEAT1 knockdown reducing human hepatocellular carcinoma cell growth through ATGL [114]. TGs are the main components of lipid droplets, and ATGL is the key enzyme in TG breakdown.

**Summary**:
**Name/organism:** NEAT1/LINC00084/MENEPSILON/BETA/NCRNA00084/TNCRNA/TP53LC15/VINC; Homo sapiens
**Databases &Code:** HGNC: 30815; NCBI Gene: 283131; Ensembl: ENSG00000245532; OMIM^®^: 612769
**Chromosomal location& size:** 11q13.1; 11: 65,422,774–65,445,540 forward strand; 9 transcripts
**Interaction with other molecules:** miR-342-3p
**Biological consequence of such interaction:** Regulation of lipid droplet aggregation; affect TG metabolism

#### 4.2.10. Nipsnap Homolog 3B

Another lncRNA, nipsnap homolog 3B (LOC286367/NIPSNAP3B) is situated in the chromosome 9q31.1. Bioinformatic analysis of differentially expressed lncRNAs and mRNAs in THP-1 macrophages revealed that *LOC286367* and *ABCA1* are located on the same chromosome but with opposite transcription directions [115]. Overexpression of LOC286367 hampers the expression of ABCA1, leading to intracellular lipid accumulation. Overexpression of ABCA1 in C57BL/6 mice was associated with an anti-atherogenic profile related to lower plasma cholesterol, cholesteryl ester, free cholesterol, and non-high-density lipoprotein cholesterol (non-HDL-C) levels, as well as elevated HDL-C, ApoA-I, and ApoE levels [116]. In turn, ABCA1 knockout in mice translated into enhanced atherosclerosis compared to control mice [117]. Therefore, it appears that targeting LOC286367 holds promise for significant improvement in the clinical outcome of atherosclerotic cardiovascular diseases.

**Summary**:
**Name/organism:** NIPSNAP3B/FP944/LOC286367/FLJ11275, SNAP1
**Databases &Code:** HGNC: 23641, NCBI Gene: 55335; Ensembl: ENSG00000165028; OMIM^®^: 608872; UniProtKB/Swiss-Prot: Q9BS92
**Chromosomal location& size:** 9q31.1; Ch9: 104,764,129–104,777,764 forward strand; 3 transcripts (splice variants), 160 orthologues and 3 paralogues
**Biological consequence of such interaction:** Putative role in vesicular trafficking; promotion of intracellular lipid accumulation

#### 4.2.11. Long Noncoding RNA Regulator of Akt Signalling Associated with HCC and RCC 

Long noncoding RNA regulator of Akt signalling associated with HCC and RCC (*lncARSR*) is situated on chromosome 9q21.31. Increased expression of this lncRNA was observed in patients with hypercholesterolaemia and mice fed a high-cholesterol diet [118]. Overexpression of lncARSR in mice led to higher lipid levels in serum and liver fragments, while lncARSR knockdown resulted in a significant decrease in plasma lipid levels in mice fed a high-cholesterol diet compared to control mice. Furthermore, lncARSR overexpression promoted the expression of HMG-CoA reductase (HMGCR), which is the rate-limiting enzyme in cholesterol synthesis. Additionally, it also enhanced the hepatic de novo cholesterol synthesis rate. The result of another study revealed that lncARSR upregulated the expression of SREBP-2, which in turn modulated the expression of cholesterol-related genes, such as HMGCR and low-density lipoprotein receptor (LDLR) [119]. These findings suggest that lncARSR promotes hepatic cholesterol biosynthesis, indicating its potential as a therapeutic target for cholesterol homeostasis disorders.

**Summary**:
**Name/organism:** LNCARSR/ lnc-TALC
**Databases &Code:** HGNC: 53864; NCBI Gene: 102723932; Ensembl: ENSG00000233086
**Chromosomal location& size:** 9q21.31; Ch 9: 79,505,804–79,567,802 reverse strand; 10 transcripts (splice variants)
**Interaction with other molecules:** SREBP-2
**Biological consequence of such interaction:** Promotion of the expression of HMG-CoA reductase (HMGCR), enhancement of hepatic de novo cholesterol synthesis rate

#### 4.2.12. LDLR Antisense RNA 1

LDLR antisense RNA 1 (*LncRNA BM450697/ LDLR-AS1*) has been recognised as a regulator of the LDLR family, members of which play significant roles in cardiovascular disease and lipoprotein homeostasis [120]. Mechanistically, BM450697 reduces LDLR transcription by hampering interactions with PolII and potentially SREBP1a (at the LDLR promoter) [121].

**Summary**:
**Name/organism:** BM450697/LDLR-AS1
Human
**Databases &Code:** HGNC: 54407, NCBI Gene: 115271120
**Chromosomal location& size:** 19p13.2; overlaps the 5′ UTR and coding sequence of the LDLR n the antisense orientation
**Interaction with other molecules:** PolII and potentially SREBP1a
**Biological consequence of such interaction:** Downregulation of the production of the low density lipoprotein receptor.

#### 4.2.13. Long Non-Coding RNA Growth Arrest-Specific 5 

Another long non-coding RNA, growth-arrest-specific 5 (*GAS5*), which is located on human chromosome 1q25.1, plays a key role in the development of atherosclerosis [89]. Significantly higher GAS5 levels have been reported in atherosclerotic plaques in affected individuals [122]. Overexpression of GAS5 hindered enhancer of zeste homologue 2 (EZH2)-mediated ABCA1 expression through histone methylation in THP-1 macrophages, thus leading to increased lipid accumulation. In *ApoE−/−* mice with atherosclerosis, it was associated with elevated levels of TC, LDL, cholesterol ester (CE), and free cholesterol (FC) [123]. Conversely, knockdown of GAS5 stimulated cholesterol reverse transport and reduced lipid accumulation by upregulating ABCA1 expression [123]. Moreover, it prevented the progression of atherosclerosis [123]. Therefore, it appears that targeting GAS5 could be a promising strategy for atherosclerosis therapy.

**Summary**:
**Name/organism:** GAS5/NCRNA00030/SNHG2; Homo sapiens
**Databases &Code:** HGNC: 16355; NCBI Gene: 60674; Ensembl: ENSG00000234741; OMIM^^®^^: 608280
**Chromosomal location& size:** 1q25.1; Ch 1: 173,858,559–173,868,882 reverse strand; 91 transcripts (splice variants)
**Interaction with other molecules:** bind to the DNA binding domain of the glucocorticoid receptor (nuclear receptor subfamily 3, group C, member 1)
**Biological consequence of such interaction:** blockage of the activation of glucocorticoid receptor, regulation of the transcriptional activity of other receptors, such as androgen, progesterone and mineralocorticoid receptors

### 4.3. LncRNA with Ambiguous Effects

#### 4.3.1. Apolipoprotein A1 and A4 Antisense RNAs 

Certain lncRNAs have been identified as modulators of lipoprotein metabolism through their spatial (positional) expression patterns. Specific lncRNAs affects apolipoproteins’ expression and function. Apolipoproteins constitute a class of lipid-binding proteins integral to lipoprotein particles, lipid transport processes, and ligands for certain cell surface receptors [25]. In plasma, apolipoprotein A1 (APOA1) represents a component of HDLs. Notably, the human apolipoprotein gene cluster, encoding APOA1, APOC3, APOA4, and APOA5, harbours two antisense lncRNAs, namely apolipoprotein A1 antisense RNA (APOA1-AS) and apolipoprotein A4 antisense RNA (APOA4-AS). These small molecules modulate the expression of associated apolipoprotein genes and plasma lipoprotein levels [12,124,125]. APOA1-AS was found to suppresses APOA1 expression via the interaction with SUZ12 which is a component of the polycomb repressive complex 2 (PRC2) involved in chromatin silencing [124]. Conversely, APOA4-AS interacts with protein HuR (Hu-antigen R, or ELAV-like protein 1) which stabilizes APOA4 mRNA. Halley et al. [124] demonstrated that the long non-coding natural antisense transcript APOA1-AS negatively regulates APOA1 expression via regulation of histone methylation patterns on chromatin regions flanking the APOA1 gene. ApoA1-AS, situated in the APO gene cluster encompassing several APO genes, influences the binding of LSD1 and SUZ12 to the APO cluster, thereby epigenetically modifying this genetic locus and inhibiting the expression of ApoA1, ApoA4, and ApoC3 [1]. Antagonizing ApoA1-AS in primates raised hepatic ApoA1 expression and circulating levels of this apolipoprotein. APOA1-AS knockdown impairs the interaction between SUZ12 and the APO gene cluster, thus reducing H3K27 trimethylation marks along the APOA1 promoter area and leading to diminished gene expression in human HepG2 hepatocytes and African green monkey primary hepatocytes [25,125]. The impact of ApoA1-AS silencing on the regulation of HDL function and lipoprotein metabolism necessitates further investigation. ApoA4-AS, has been identified in the liver of obese mice [1,125]. The expression of apolipoprotein A4 is regulated by APOA4-AS which stabilize apolipoprotein A4 mRNA in mouse liver via direct interaction with HuR [25,125]. Upregulated in human samples from subjects with fatty liver, ApoA4-AS displays a similar expression pattern to the ApoA4 gene. Intriguingly, circulating ApoA4 levels correlate with HDL plasma levels. Direct interaction of ApoA4-AS with HuR enhances ApoA4 mRNA stability [125]. HuR is an RNA-binding protein that regulates mRNA stability and translation. In vivo, the silencing of ApoA4-AS did not exert impact on hepatic triacylglycerol (TAG) content but significantly decreased plasma TAG and cholesterol levels. The mechanism by which ApoA4-AS influences circulating lipids requires clarification, however, it was suggested to possibly affect VLDL-C secretion via reducing hepatic ApoA4 levels [125]. Given the specificity of APOA4-AS in regulating apolipoprotein A4, it could be considered as a potential therapeutic target for metabolic diseases. Subsequent studies are warranted to unravel the intricate mechanisms underlying ApoA4-AS control of lipoprotein metabolism and hepatic lipid homeostasis [125].

**Summary**:
**Name/organism:** ApoA1-AS; Homo sapiens
**Databases &Code:** GeneCaRNA, HGNC: 40079, NCBI Gene: 104326055, Ensembl: ENSG00000235910, OMIM^®^: 620112
**Chromosomal location& size:** 11q23.3, Size: 20,898 bases, Orientation: Plus strand
**Interaction with other molecules:** SUZ12, a component of the polycomb repressive complex 2 (PRC2)
**Biological consequence of such interaction:** Suppression of APOA1 expression
**Name/organism:** ApoA4-AS; mouse
**Databases &Code:** Ensembl and UCSC Genome Database
**Chromosomal location& size:** ∼900-nt
**Interaction with other molecules:** APOA4
**Biological consequence of such interaction:** APOA4-AS may regulate the expression of APOA4

#### 4.3.2. lncRNA Induced by HCV, Regulator of SREBF1

The long noncoding RNA HCV-regulated 1 (*lncHR1*) that is localized in both the nucleus and cytoplasm has been demonstrated to negatively impact lipid metabolism by suppressing the expression of the SREBP-1c gene [34]. Hepatitis C virus (HCV) infection is known to trigger SREBP expression and hepatic accumulation of lipids [1,55]. LncHR1 regulates the expression of SREBP-1-responsive genes. Overexpression of lncHR1 was found to reduce the expression of SREBP-1 and Fatty Acid Synthase (FAS) as well as to decrease the formation of lipid droplet in Huh7 cells. In turn, silencing of lncHR1 in Huh7 cells was associated with increased SREBP-1 and FAS expression and stimulated lipid droplet accumulation [55]. These findings were further corroborated in vivo using a transgenic mouse model overexpressing lncHR1 (lncHR1TG). Consistent with the in vitro results, hepatic expression of SREBP-1 and FAS was diminished in lncHR1TG mice compared to wild-type (WT) mice. Notably, plasma TG levels were also diminished in the transgenic mice compared to WT mice [55]. However, the precise mechanism of lncHR1-related regulation of circulating lipids remains not clear. Therefore, there is a need for further studies which would evaluate the impact of circulating TGs on VLDL production, catabolism, or clearance.

**Summary**:
**Name/organism:** LNCHR1; Homo sapiens
**Databases &Code:** Ensemble: ENSG00000257400.1; HGNC:56254
**Chromosomal location& size:** 12q22: 94,491,546–94,496,442 reverse strand; Size: 420 bp
**Interaction with other molecules:** SREB-1c
**Biological consequence of such interaction:** Regulation of the expression of SREBP-1-responsive genes

#### 4.3.3. Solute Carrier Family 25 Member 15 (SLC25A15/lnc-HC)

The long non-coding RNA Lnc-HC is expressed in both the liver and adipocytes where it exerts PPARγ-mediated regulatory control over the expression of key enzymes in the cholesterol biosynthesis pathway, namely cholesterol 7α- hydroxylase (CYP7a1), and the cholesterol efflux transporter ABCA1 via miR-130b-3p [1,126,127]. Lnc-HC inhibition is associated with significant increase in the expression of both CYP7a1 and ABCA1 in hepatocytes. Lnc-HC was found to form a complex with RNA binding protein - hnRNPA2B1, which interacts with CYP7a1 and ABCA1, thereby regulating the expression of both transcript and subsequently modulating hepatic cholesterol catabolism [126].

Also, Lan et al. [127] reported that lnc-HC plays a negative role in hepatic cholesterol catabolism. In an 8-week high-fat diet-induced hepatic lipid disorder model, suppression of lnc-HC significantly increased serum levels of TG and HDL cholesterol, suggesting that lnc-HC knockdown could be a potential therapy for lipid metabolism disorders. However, in the liver model, lnc-HC suppression led to a notable increase in TG concentrations and a vacuolar-like steatosis phenotype. These findings indicate that lnc-HC has multiple functions in systemic lipid metabolism, including beneficial effects in treating dyslipidaemia but also causing adverse effects in hepatic lipid metabolism. In vitro studies revealed that lnc-HC knockdown significantly enhanced the expression of genes involved in free fatty acid (FFA) uptake, such as *Fatp-1* (fatty acid transport protein 1) and aP2 (adipocyte protein 2), and those related to TG synthesis, including *Acc2* (acetyl-CoA carboxylase beta), *Acsl1* (acyl-CoA Synthetase Long Chain Family Member 1), and *Dgat2* (diacylglycerol O-acyltransferase 2). However, it has minimal impact on genes related to hepatocytic very VLDL formation, such as *Dgat1* (diacylglycerol O-acyltransferase 1) and Mttp (microsomal triglyceride transfer protein). In vivo, lnc-HC exerted little effect on serum LDL-C levels, which are primarily derived from TG-enriched VLDL-C. This explains the observed increase in hepatic TG and decrease in serum TG [127]. Additionally, lnc-HC negatively modulates the mitochondrial FFA β-oxidation gene *Cpt-1α* (carnitine palmitoyltransferase 1A), implying its potential involvement in FFA consumption and utilization. Subsequent studies confirmed that lnc-HC promotes FFA uptake, TG synthesis, and lipid droplet accumulation in cells. It appears that following FFA treatment, upregulated lnc-HC protects hepatocytes from excessive expressing PPARγ, thereby helping to avoid excessive lipid droplet formation and lipid accumulation in the liver. Subsequent experiments are crucial to elucidate whether these regulatory mechanisms are operational in an in vivo context.

**Summary**:
**Name/organism:** lnc-HC/SLC25A15/HHH/ORC1/ORNT1/D13S327
**Databases &Code:** GenBank: MN026163.1
**Chromosomal location& size:** 1063 bp, linear
**Interaction with other molecules:** Coregulator: hnRNPA2B1
**Biological consequence of such interaction:** Reduction of the stability of mRNAs encoding Cyp7a1 and Abca1 (critical enzymes that contribute to cholesterol catabolism).

#### 4.3.4. Metastasis-Associated Lung Adenocarcinoma Transcript 1 

Long non-coding RNA metastasis-associated lung adenocarcinoma transcript 1 (lncRNA *MALAT1*) was demonstrated to play a role in atherosclerosis pathogenesis [128]. It is also a biomarker in various cancers [128]. MALAT1 play a dual role in atherosclerosis. Its silencing alleviates oxidized low-density lipoprotein (ox-LDL)-induced endothelial inflammation and protects the endothelium from oxidative stress [129]. Moreover, it has been suggested that MALAT1 influences lipid metabolism disorders, inflammatory responses, and progression of atherosclerosis by regulating ox-LDL-related macrophages [130]. MALAT1 has been found to target ABCA1 through multiple mechanisms, contributing to cholesterol efflux via the MALAT1-miR-17-ABCA1 axis. Silencing MALAT1 leads to increased cholesterol accumulation in ox-LDL [130]. Overexpression of MALAT1 reduces viability and promotes apoptosis in ox-LDL-treated vascular smooth muscle cells (VSMCs). Additionally, MALAT1 was demonstrated to regulate cardiac inflammation and stimulate acute myocardial infarction through interaction with miR-125b, which is a mediator in several vascular diseases [131,132]. miR-125b decreases endothelin-1 expression, inhibits vascular endothelial-cadherin mRNA translation, and lowers endothelial permeability, all of which are involved in the development of atherosclerosis [132,133]. Lv et al. [128] reported higher levels of lncRNA MALAT1 in patients with CVD compared to controls. In these patients, greater expression of MALAT1 accompanied by low miR-125b levels were associated with more severe coronary stenosis, hyperlipidaemia, and systemic inflammation as well as more frequent occurrence of major adverse cardiac events (MACE). MALAT1 also promotes the upregulation of inflammatory molecule C-reactive protein (CRP) which contributes to the development of atherosclerotic plaque. Moreover, it triggers several signalling pathways, including p38 MAPK/nuclear factor-kappa B (NF-κB) that are involved in cardiac dysfunction and CHD [134]. MALAT1 acts as a miRNA-124-3p sponge thus affecting PPARα expression.

**Summary**:
**Name/organism:** MALAT1; HCN, LINC00047, MASCRNA, NCRNA00047, NEAT2, PRO1073; Homo sapiens
**Databases &Code:** Ensembl: ENSG00000251562.11; HGNC:29665
**Chromosomal location& size:** 11q13.1: 65,497,640–65,508,073 forward strand; 66 transcripts (splice variants)
**Interaction with other molecules:** miR-17-ABCA1; miRNA-124-3p (sponge)
**Biological consequence of such interaction:** Contribution to cholesterol efflux, promotion of the upregulation of inflammatory CRP, modulation of PPARα expression.

#### 4.3.5. A Novel Long Non-Coding RNA in Lipid Associated Single Nucleotide Polymorphism Gene Region 

LASER (a novel long non-coding RNA in Lipid Associated Single nucleotide polymorphism gEne Region) is located on chromosome 11q12 [69]. A positive correlation between LASER expression and cholesterol levels has been demonstrated [69]. LASER expression is high in hepatocytes as well as peripheral mononuclear cells (PBMCs). Li et al. [135] reported that knockdown of LASER using small interfering RNA (siRNA) resulted in improved intracellular cholesterol levels, Moreover, it enhanced the expression of cholesterol metabolism genes at both protein and mRNA levels by suppressing proprotein convertase subtilisin/kexin 9 (PCSK9) expression. PCSK9, a key regulator of cholesterol homeostasis primarily secreted from the liver, increases circulating LDL-C concentrations in the bloodstream [136].

**Summary**:
**Name/organism:** LASER/ LINC02702; Homo sapiens
**Databases &Code:** HGNC:54217; Ensembl: ENSG00000237937; NCBI Gene: 101929011
**Chromosomal location& size:** 11q23.3; 11: 116,639,422–116,658,295 forward strand; 4 transcripts
**Interaction with other molecules:** probably PCSK9
**Biological consequence of such interaction:** Enhancement of the expression of cholesterol metabolism genes

The summary of selected lncRNAs roles in lipid metabolism is presented in Table 1.

## 5. LncRNAs as Diagnostic and Therapeutic Targets in Lipid Disorders

Nowadays, diagnosing cholesterol-related diseases involves measuring lipid fractions, such as HDL, LDL, TG, and total cholesterol, in the blood [127,138]. However, this method requires 9 to 12 h of fasting for accurate results and provides limited information; therefore, there is a need for more accurate diagnostic measures [139]. Currently, lncRNAs are being developed as diagnostic and prognostic biomarkers to enable more accurate and detailed diagnosis [140,141]. They also hold potential as new targets for disease treatment [142]. Preclinical and human studies highlight their characteristics, including stability, high cell-type and tissue specificity, disease specificity, and ease of detection, making them suitable materials for patient diagnostics [68]. Additionally, lncRNAs may offer advantages over proteins due to their lower translation, rapid turnover, and low expression levels, potentially allowing for faster effects with lower doses. The deregulation of many lncRNAs, including RAPIA [105], H19 [143], CASC11 [144], GAS5 [145], TUG1 [146], MIAT [95], lnc00113 [147], and NEXN-AS1 [148], has been observed in patients with atherosclerosis [69]. The level of some lncRNAs, such as CASC11, H19, TUG1, and MIAT, can be analysed in serum samples as potential diagnostic markers for atherosclerosis. Other lncRNAs, such as MIR22HG, AL117190.1, LINC00184, COL4A2-AS1, and MEG3, could serve as important prognostic markers for patients [149]. The lncRNA CHROME, participating in cholesterol efflux and HDL biogenesis, was higher in the plasma and atherosclerotic plaques, thus it was suggested to be a novel biomarker for CAD progression [70]. In turn, plasma LeXis, which is involved in cholesterol metabolism and hepatic steatosis development, was recognised as a non-invasive diagnostic biomarker for NASH [150]. It appears that developing lncRNA biomarkers for diagnosing lipid-related diseases is promising.

The best approach to prevent and manage lipid-related diseases is to adopt lifestyle modifications, for example, to increase exercise and shift to a healthy diet [68,151]. Patients with chronically high lipid levels require treatment to lower them; thus, the primary aim of treatment is to decrease cholesterol to suitable levels. Generally, RNA interference (RNAi), based on short-hairpin RNA (shRNA), siRNA, or antisense oligonucleotides (ASOs) or small-molecule inhibitors, seems a promising approach for targeting lncRNA silencing [152]. This method has been found to be effective at the cellular level and in animal models [153,154]. Oligonucleotide therapeutics found application in the treatment of various diseases, including cancer, liver, and kidney diseases, infectious diseases, as well as atherosclerosis [68,155,156]. Currently, the most advanced therapies based on lncRNA-targeting utilise ASOs, which are short, single-stranded DNA sequences, complementary to unique RNA sequences. After binding with specific RNA, ASOs lead to premature transcription termination and inhibition of lncRNA expression [157,158]. However, clinical use of ASOs is limited due to their in vivo toxicity and the lack of efficient delivery systems to cells and tissues. To ameliorate their pharmacological properties, modifications have been introduced to ASO technology to improve efficacy, potency, and stability, increase resistance to nuclease degradation, as well as limit non-specific immune responses [68,158]. So far, several mRNA-therapeutic ASOs have been approved by the Food and Drug Administration (FDA) and the European Medicines Agency (EMA), and many more are at late-stage clinical development, including some ASOs targeting lncRNA-dependent lipid-related diseases [159,160]. Lentiviral shRNA targeting lncRNA myocardial infarction-associated transcript (MIAT) has been demonstrated to considerably hamper atherosclerosis progression and enhance plaque stability in vivo [94]. In addition to targeting lncRNA directly, controlling lncRNA function by inhibiting its interaction with RNA-binding proteins has also been explored [15,161]. RNA interference therapeutics have recently advanced from preclinical development to clinical trials [162]. However, the development of effective and safe delivery systems is crucial for their clinical application.

Small molecules are also extensively used for targeting various diseases therapeutically. These compounds have better cellular uptake and fewer administrative challenges than ASOs and viral vectors for RNAi delivery. Small-molecule inhibitors target lncRNAs by preventing them from binding to their RNA-binding proteins.

In vivo targeting of Nudt6 (which is an antisense transcript of fibroblast growth factor 2; FGF2) with ASO, using murine and porcine models of carotid artery disease and abdominal aortic aneurysm, showed limited disease progression [163]. Nudt6 knockdown restored FGF2, improving vessel wall morphology and fibrous cap stability. In turn, in vitro, overexpression of NUDT6 impaired SMCs’ migration, limited their proliferation, and increased apoptosis. Cysteine- and glycine-rich protein 1 (CSRP1) was identified as an alternative direct NUDT6 interaction molecule, which controls cell motility and SMC differentiation. Therefore, it appears that NUDT6 silencing could be a novel RNA-based therapeutic strategy for vascular diseases [163].

## 6. Conclusions

The understanding of the role of various lncRNAs remains poor; thus, extensive efforts are required to characterise already identified lncRNAs. Challenges arise due to the poor conservation of lncRNAs across species and the diverse mechanisms through which they impact cellular functions. The role of lncRNAs in lipid metabolism and other biological processes emphasises the ongoing necessity to unravel mechanisms related to lncRNAs to possibly reduce the risk of lipid disorders, atherosclerosis, and other diseases. Progress in this field is advancing rapidly, notwithstanding the hurdles associated with the dysregulation of lncRNAs in various disease states. The identification of dysregulated lncRNAs may pose as promising candidates for therapeutic interventions, since strategies enabling the restoration of their levels could offer an effective means to impede disease progression without disrupting normal biological functions.

Both deficiency and accumulation of cellular cholesterol are the characteristic traits of some lipid-related diseases. Therefore, maintaining cholesterol homeostasis is of high importance across various cell lines. In humans, multiple feedback and compensatory mechanisms were found to modulate cholesterol homeostasis. LncRNAs have emerged as key regulators of lipid metabolism and the progression of lipid-related diseases. Their discovery has provided novel biomarkers and therapeutic targets for patients with lipid-related diseases.

While lncRNAs often exhibit tissue- and disease-specific expression patterns, they are less conserved than protein-coding genes. This lack of conservation positions lncRNAs as potentially superior therapeutic targets for disease diagnoses and prognoses compared to protein-coding genes. Their status as biologically functional molecules further suggests that their expressions may serve as valuable biomarker candidates for various disease states. Despite these promising aspects, much remains unknown about the functions of most lncRNAs, necessitating extensive studies to elucidate their roles in physiology, development, and disease. Understanding the pharmacokinetics and toxicity of lncRNAs is crucial for their effective use as therapeutic targets. The unique biological characteristics of these regulatory RNAs offer potential novel treatment options.

## Figures and Tables

**Table 1:** The summary of the roles of selected lncRNAs in lipid metabolism.

lncRNA	Impact on Lipid Metabolism and Cardiovascular Risk	Refs.
lncRNA H19	− Stabilisation of the SREBP-1c protein − Promotion of hepatic lipogenesis	[55]
AC068234.2–202	− Cis-regulator of ITGB3-stimulation of platelet activation − Involved in the pathogenesis of CAD	[34]
AP001033.3–201	− Trans-regulator of TBXA2R-stimulation of platelet activation − Involved in the pathogenesis of CAD	[34]
ApoA1-AS	− Suppression of APOA1 expression via the interaction with SUZ12 − Negative regulation of APOA1 expression by modulation of histone methylation patterns on chromatin regions flanking the APOA1 gene	[124]
ApoA4-AS	− Stabilisation of APOA4 mRNA via interaction with protein HuR − Silencing significantly decreased plasma TAG and cholesterol levels − Plausible impact on VLDL secretion via reducing hepatic ApoA4 levels	[125]
Overexpression of ENST00000602558.1	− Downregulation of ABCG1 mRNA, decreased protein levels, and reduced ABCG1-mediated cholesterol efflux to HDL from VSMC − Promotion of lipid accumulation and increased total cholesterol/triglyceride (TC/TG) levels	[89]
RP5-833A20.1	− Modulation of cholesterol homeostasis, inflammatory responses, and foam cell formation through its interaction with miRNA (miR-382-5p) − Increases HDL-C and reduces levels of LDL-C and VLDL-C, leading to regression of atherosclerosis in ApoE−/− mice	[23,59]
LeXis	− Mitigates cholesterol synthesis in situations of excessive cholesterol availability by impeding the recruitment of RNA polymerase II to Srebf2 and its transcriptional targets − Suppresses the expression of genes related to cholesterol biosynthesis and hepatic cholesterol content	[12,60]
MeXis	− Specific regulator of LXR-induced ABCA1 expression—amplifies Abca1 expression, enhancing macrophage cholesterol efflux − Promotes effective cholesterol elimination and considerably decreases the risk of atherosclerosis	[21,61,62]
LncHR1	− Reduces expression of SREBP-1 and fatty acid synthase (FAS) − Reduces lipid droplet formation − Decreases hepatic and circulating TG levels	[55]
RP1-13D10.2	− Promotes increased expression of LDL receptors − Enhances plasma LDL-C internalisation − Reduces media levels of apolipoprotein B − Possible involvement in statin-induced reduction of LDL-C levels	[24,63]
LncLSTR	− Enhances ApoC2 expression and, consequently, stimulates LPL − Involved in systemic TG metabolism—regulates TG clearance	[33]
Lnc-HC	− Regulates CYP7a1 and ABCA1 expression − Plays a negative role in hepatic cholesterol catabolism − Potentially involved in FFA consumption and utilisation − Protects hepatocytes from excessively expressing PPARγ, thus helping to avoid excessive lipid droplet formation and lipid accumulation in the liver	[1,126,127]
LincRNA-DYNLRB2-2	− Increases the expression of ABCA1 and GPR119 − Facilitates cholesterol efflux and reductions in neutral lipid accumulation	[58]
CHROME (PRKRA-AS1)	− Upregulated in the plasma and arterial plaques of patients with CVD − Stimulation of cholesterol secretion and HDL synthesis via suppressing specific miRNAs (miR-27b, miR-33a/b, and miR-128)	[25,69,70]
lncRNA LIPTER	− Helps with lipid droplet transport within cardiomyocytes − Alleviates cardiomyopathies and preserves cardiac function in models of obesity and diabetes	[71,73]
lncRHPL	− Supresses hepatic VLDL secretion by modulating the hnRNPU/BMAL1/MTTP axis without affecting lipogenesis or fatty acid oxidation − Involved in pathogenesis of dyslipidaemia and NAFLD	[78]
LncNONMMUG027912	− Involved in cholesterol metabolism − Reduces lipid accumulation, TG levels, and expression of lipid biosynthesis genes − Limits liver inflammation in non-alcoholic fatty liver disease (NAFLD)	[79]
MALAT1	− Regulates ox-LDL-related macrophages − Regulates cardiac inflammation and stimulates acute myocardial infarction through interaction with miR-125b − Involved in atherosclerosis pathogenesis − Promotes the upregulation of inflammatory CRP − Stimulates hepatic lipogenesis	[134,135,136,137,138,139]
TUG1	− Involved in glucose homeostasis, insulin signalling, and lipid metabolism − Decreases expression of ApoM, ABCA1, and ABCG1, consequently reducing the rate of cholesterol efflux	[90]
MIAT	− Increases lipid content and blood lipid levels, stimulates the formation of atherosclerotic plaques, and reduces collagen content in the plaques − Enhances angiogenesis and increases the expression of inflammatory factors (IL-1β, IL-6, and TNF-α) in an atherosclerosis model	[95,103]
LncRNA RP11-728F11	− Stimulates CD36 expression and intracellular cholesterol accumulation − Contributes to the development of atherosclerosis	[100]
lncRNA RP5-833A20.1	− Involved in lipid homeostasis and adipocyte differentiation	[25]
lncRNA ANRIL	− Involved in atherosclerosis	[84]
LASER	− Enhances the expression of cholesterol metabolism genes	[136]
lncRNA ENST00000416361	− Reduces the levels of the inflammatory factors IL-6 and TNF-α and thus CAD progression − Affects lipid metabolism-related genes	[101,104]
LncRNA RAPIA	− Associated with the progression of atherosclerosis − Regulates the proliferation and apoptosis of macrophages	[105,106]
NEAT1	− Regulates lipid droplet aggregation − Involved in atherosclerosis development − Increases TNF-α, IL-1β, and IL-18 levels, and exacerbates ischemia–reperfusion injury − Influences TG and modulates ATGL expression	[107,109,112,113,114]
LOC286367	− Reduces the expression of ABCA1, leading to intracellular lipid accumulation	[115]
HOXC-AS1	− Decreased expression in human atherosclerotic plaques − Limits ox-LDL-induced cholesterol accumulation	[85]
LncARSR	− Increased expression in patients with hypercholesterolaemia − Increases lipid levels in serum and liver fragments − Promotes the expression of HMGCR—a rate-limiting enzyme in cholesterol synthesis − Promotes hepatic cholesterol biosynthesis	[119,128]
BM450697	− Regulator of the LDLR family − Plays important roles in cardiovascular disease and lipoprotein homeostasis	[120]
GAS5	− Involved in the development of atherosclerosis − Increases lipid accumulation	[68,123]

ATGL, adipose triglyceride lipase; CRP, C-reactive protein; CYP7a1, cholesterol 7α-hydroxylase; HMGCR, HMG-CoA reductase; LDL-C, low-density lipoprotein cholesterol; LDLR, low-density lipoprotein receptor; LPL, lipoprotein lipase; NAFLD, non-alcoholic fatty liver disease; TG, triglyceride; VLDL-C, very-low-density lipoprotein cholesterol.

## Data Availability

Not applicable.
